# Adaptive Optics Technology for High-Resolution Retinal Imaging

**DOI:** 10.3390/s130100334

**Published:** 2012-12-27

**Authors:** Marco Lombardo, Sebastiano Serrao, Nicholas Devaney, Mariacristina Parravano, Giuseppe Lombardo

**Affiliations:** 1 Fondazione G.B. Bietti IRCCS, Via Livenza 3, 00198 Rome, Italy; E-Mails: serrao@serraolaser.it (S.S.); criparra@tin.it (M.P.); 2 Applied Optics Group, School of Physics, National University of Ireland, Galway, Ireland; E-Mail: nicholas.devaney@nuigalway.ie; 3 CNR-IPCF Unit of Support of Cosenza, c/o University of Calabria, Ponte P. Bucci Cubo 31/C, 87036 Rende, Italy; E-Mail: giuseppe.lombardo@cnr.it; 4 Vision Engineering, Via Adda 7, 00198 Rome, Italy

**Keywords:** adaptive optical systems, optical sensors, biomedical imaging techniques, eye

## Abstract

Adaptive optics (AO) is a technology used to improve the performance of optical systems by reducing the effects of optical aberrations. The direct visualization of the photoreceptor cells, capillaries and nerve fiber bundles represents the major benefit of adding AO to retinal imaging. Adaptive optics is opening a new frontier for clinical research in ophthalmology, providing new information on the early pathological changes of the retinal microstructures in various retinal diseases. We have reviewed AO technology for retinal imaging, providing information on the core components of an AO retinal camera. The most commonly used wavefront sensing and correcting elements are discussed. Furthermore, we discuss current applications of AO imaging to a population of healthy adults and to the most frequent causes of blindness, including diabetic retinopathy, age-related macular degeneration and glaucoma. We conclude our work with a discussion on future clinical prospects for AO retinal imaging.

## 1. Introduction

The human eye functions as an optical system whose purpose is to bring the outside world into focus on the retina, thereby allowing us to see. However, the optics and how they are aligned are not perfect and the consequence is that incoming light rays deviate from the desired path that reaches the foveal center or foveola. These deviations are defined as optical aberrations [1]. Aberrations in the eye optics not only blur images formed on the retina, thus impairing vision, but also blur images taken of the retina by ophthalmic imaging cameras.

Ocular aberrations can be classified into low-order (LOA) and high-order aberrations (HOA). Low-order aberrations, such as defocus and astigmatism, are the predominant optical aberrations and they account for approximately 90% to the overall wavefront aberration (WA) of the eye. Although HOA make a small contribution (on average ≤ 10%) to the total variance of the eye WA, their effect on image quality is well known as well as the fact that their correction can significantly improve visual performance and retinal imaging [2–4].

Diagnosis of retinal diseases at an early stage is crucial for the treatment and avoidance of serious visual loss. Across the developed world, the major causes of vision loss can be attributed to age-related macular degeneration, diabetic retinopathy and glaucoma [5]. Diagnosis usually occurs once damage has already happened to some extent and this is due to the relatively poor resolution of current retinal imaging techniques which limits their ability to detect abnormalities of the retinal microstructures, including photoreceptors cells, capillaries and nerve fiber bundles. Imaging modalities able to detect and monitor pathological variations of the retinal microstructures at a pre-clinical stage of disease represent the basis for designing new diagnostic and treatment protocols to preserve the normal integrity and function of the retina. The need for sensitive and accurate diagnostic tools is increasing as a growing number of new treatment options become available to ophthalmologists, including gene therapy, nano-targeted drug delivery devices and micro-pulsed lasers.

Optical imaging is the preferred method to non-invasively investigate the retina and to perform efficient retinal therapy. Retinal cameras for wide field clinical imaging are generally designed without considering aberration correction beyond defocus, the resolution at which images could be recovered from the retina *in vivo* has been limited to the macroscopic scale. In order to bring the lateral resolution of ophthalmoscopes to the microscopic scale, it is necessary to compensate not only for defocus, but also astigmatism and higher order aberrations. Adaptive optics (AO) is a technology used to improve the performance of optical systems by minimizing aberrations. It has been developed for astronomical telescopes in order to remove the effect of atmospheric turbulence from astrophysics objects and only in recent years has been extended to ophthalmology [6,7]. When applied to the optical system of the eye, AO technology can provide substantial improvements in the sharpness of retinal images that are normally degraded by ocular aberrations [8]. AO retinal imaging can theoretically improve the lateral resolution to 2 μm and therefore provide information about the retinal microstructures that cannot be obtained with current retinal imaging techniques.

The scope of this review is to describe some of the technical aspects that have made AO retinal imaging in human eyes possible and to discuss applications and future prospects for noninvasive clinical imaging of retinal diseases with microscale level of resolution.

## 2. The Optical System of the Human Eye

**Figure 1.**
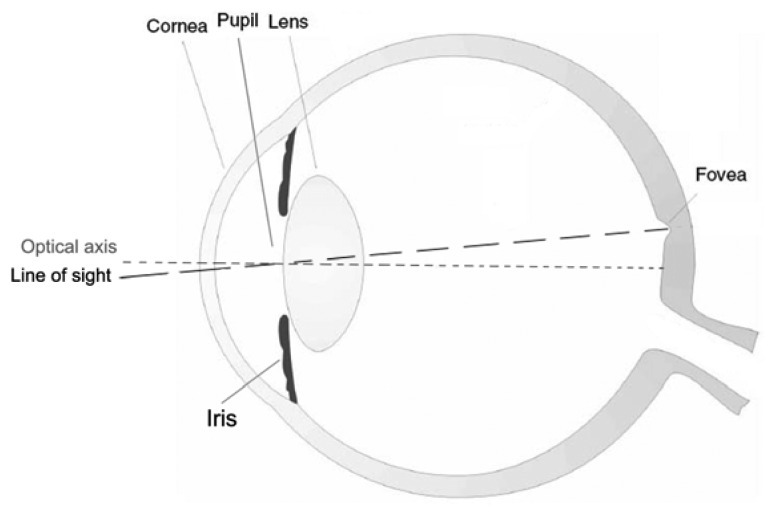
The optical system of the human eye consists of three main components, *i.e.*, the cornea, the crystalline lens and the iris. The iris controls the amount of light coming into the retina by regulating the diameter of the pupil. Therefore, the pupil of the eye acts as the aperture of the system. The optical axis of the eye (dotted grey line) is defined as the line joining the centers of curvature of all the optical surfaces. However, the appropriate and convenient axis that should be used for describing the optical system of the eye is the line of sight (dashed black line), which is defined as the ray that passes through the center of the entrance pupil and strikes the center of the fovea (*i.e.*, the foveola).

The eye's optical system consists of three main components: the cornea, the crystalline lens and in between them the iris (Figure 1). The cornea, the outermost optical element, is responsible for about 2/3 of the optical power and aberrations of the eye. The iris controls the amount of light coming into the retina by regulating the pupil diameter. As in any optical system, the size of the pupil has important consequences for image formation: a smaller pupil increases the depth of focus and minimizes the effects of high-order aberrations. Conversely, the magnitude of aberrations increases with pupil dilation leading to a decrease in both visual performance and optical quality of the retinal image [1]. The crystalline lens accounts for about 1/3 of the optical power of the eye but it is capable of changing its focusing properties: controlled changes in the shape and thickness of the crystalline lens allow the eye to accommodate, the process by which the eye focuses on near objects. Even in the normal eye, departures from ideal focus (*i.e.*, aberrations) exist and degrade the eye's optical performance [8–10]. LOA are the predominant optical aberrations (90% of the overall WA of the eye): defocus (positive or negative; *i.e.*, hyperopia and myopia respectively) is the dominant aberration, followed by astigmatism (orthogonal or oblique). It is well known that the human eye suffers from HOA that cannot yet be accurately corrected and that they greatly diminish the overall optical quality of the eye, though their contribution to the overall WA of the eye is ≤10% [3,8]. The presence of HOA, beyond defocus and astigmatism, has been known by researchers since the 19^th^ century, but only in the 1990s have wavefront sensors been developed to allow routine estimation of the eye's WA. The development of ocular wavefront sensors has allowed rapid, accurate and objective measurements of wave aberrations and made large population studies possible [2,9–11]. Several authors [12–15] have measured the distribution and contribution of both LOA and HOA to the overall WA of the eye: between HOA, the magnitude of 3^rd^ order coma-like aberrations (vertical coma, horizontal coma, oblique trefoil and horizontal trefoil) and spherical aberration is higher than other higher aberration modes [1]. The eye's WA is not static but fluctuates over time: the eye's focus exhibits fluctuations about its mean value for steady-state accommodation with amplitudes ranging between 0.03 and 0.5 diopters (D). In addition, a general tendency for spherical aberration to change in a negative direction with increase in accommodation (–0.04 μm/D for accommodative levels of 1.0 to 6.0 D) has been measured, while the other HOA are not significantly influenced by accommodation [16,17]. The largest source of temporal short-term instability (seconds and minutes) of HOA is then due to the micro-fluctuations in the accommodation of the lens: the anterior curvature increases centrally and flattens peripherally during accommodation, while at the same time, the lens thickness increases and the equatorial diameter decreases. These factors may contribute to the change in the measured aberrations. Another source of fluctuations is local changes in the tear film thickness over the cornea, due to evaporation and/or blinking [1,18]. If considering a long period of time (over the course of the day and between successive days), the WA of the eye has been demonstrated to be sufficiently stable, with no significant changes in the magnitude and contributions of HOA [1,17]. An AO ophthalmic device can measure and correct for the fluctuations of the eye's WA, thus improving the resolution of images taken from the retina of patients.

## 3. Adaptive Optics Technology for Retinal Imaging

The history of adaptive optics for ophthalmic imaging is just over 15 years old. AO was first used by Dreher *et al.* in 1989 [19], but the correction was limited to only second order optical aberrations of the eye. In 1997, AO technology was successfully applied to high resolution imaging in the human eye by Liang *et al.*[20]. Since that time AO technology has advanced dramatically, including the integration of AO into different imaging modalities and improved optical design [21]. Almost all existing ophthalmic modalities have incorporated AO to enhance quality and resolution of the retinal images: flood illumination fundus imaging, confocal scanning laser ophthalmoscopy and ophthalmic optical coherence tomography, with each offering different benefits [6,7,22]. Current AO flood-illumination cameras incorporate a superluminescent diode to illuminate the retina, used in conjunction with a high-speed scientific-grade CCD. These improvements permit faster modulation (higher frame rates) and higher efficiency (shorter exposure durations) than former prototypes. Confocal scanning laser ophthalmoscopy (cSLO) and ophthalmic optical coherence tomography (OCT) are typically realized by raster scanning a focused point source across the retina. The cSLO uses the same optical path for scanning the laser spot on the retina and for delivering the returning light to the detector. A confocal pinhole just in front of the light detector collects most of the light from the retina while rejecting light originating from unwanted planes away from the image retinal plane. AO-OCT provides a high-resolution cross-sectional view through the living retina, comparable to a histological section. Adaptive optics provides three technical benefits for OCT that improve the visualization and detection of microscopic structures in the retina: (1) increased lateral resolution, (2) reduced speckle size (granular artifacts), and (3) increased sensitivity to weak reflections.

**Figure 2.**
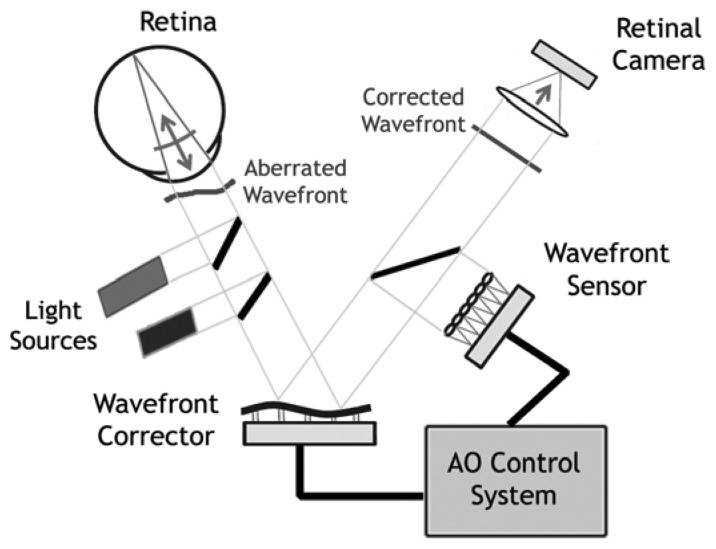
Basic layout of an adaptive optics system for retinal imaging. The system measures the ocular aberrations with a wavefront sensor and corrects for them with a wavefront corrector to achieve high lateral resolution imaging. Two light sources are generally used by an AO system: one is used to measure and correct the wavefront aberration of the eye; the second source is used to illuminate the retinal field being imaged. The AO compensated retinal image is captured by a high-resolution imaging camera.

Adaptive optics by itself does not provide a retinal image, rather an AO subsystem must be incorporated into an imaging device. A typical AO retinal imaging camera has three principal components: a wavefront sensor, a corrective element and a control system. The wavefront sensor and corrector measure and correct the eye's wave aberrations respectively. The AO controller, programmed with a computer, controls the interaction between the wavefront sensor and the corrector element; it interprets the wavefront sensor data and computes the appropriate wavefront corrector drive signals. AO systems operating in closed-loop (Figure 2) place the wavefront sensor after the wavefront corrector. In this configuration, the measured wavefront is the error signal that gets fed back to the controller to further reduce the residual aberrations in the next iteration, theoretically correcting the retinal images up to the diffraction limit.

The benefit of AO for noninvasive retinal imaging has been clearly shown with previous high-resolution images of cone photoreceptors in normal subjects [20] and with the discovery of differences in the pattern of the cone mosaic in colour blind eyes with respect to controls [23]. Since then, the use of AO retinal imaging to detect and monitor retinal abnormalities has clearly been shown in studies where high resolution images of the cone mosaic were acquired in normal eyes and eyes with retinal diseases [24]. The early diagnosis of retinal diseases and the monitoring of treatment efficacy at a cellular level provides promising clinical applications of AO technology.

Continuing efforts in this fascinating area of research now promote the design and development of innovative systems and devices for improving resolution and contrast of AO retinal images [21]. Although different methods have been proposed for measuring aberrations, the Hartmann-Shack wavefront sensor is still most widely used. Regarding the aberration correctors, many different devices have been proposed and demonstrated for ophthalmic applications. In this review, emphasis is placed on principles rather than details of individual instruments, most of which can be found in the specialized literature [25].

## 4. Wavefront Sensing Technology

Several wavefront sensing techniques have been developed for the measurement of the wavefront error in human eyes. Most wavefront sensors are based on the same principle, which is an indirect measurement of the first or second derivatives of the wavefront at the location of the pupil plane of the eye and the reconstruction of the complete wavefront by integrating these derivatives. In general, the apparatus directs a small beam of light into the eye which then backscatters off the retina. The scattered light leaving the eye gets aberrated by the eye's optics before being recorded by the imaging components of the wavefront sensor.

The Shack-Hartmann (S-H) type wavefront sensor is the most common method for measuring ocular aberrations. The device was introduced in clinical ophthalmology in 1994 [26,27] and uses an array of micro-lenslets each of which observes the out-going beam at a different pupil location. The resulting light, aberrated by the eye's optics, is focused as an array of spots onto a detector. The best estimate of each spot's position and how far it deviates from a reference is calculated. The result is proportional to the average wavefront slope at that particular location of the pupil. Detailed information on the principle of S-H wavefront sensing can be found in previous work [25]. Limitations of the method include the quantity of light needed, the dynamic range and the fact that analysis of data from a S-H sensor does not consider the quality of the individual spots formed by the lenslet array [25]. However, experience has shown that the quality of spot images can vary greatly over the pupil of a human eye. If the wavefront shape within a single lenslet varies significantly, the spot pattern formed by that lenslet will be blurred making it more difficult to estimate its location. In the context of wavefront sensing, the term dynamic range represents the maximum wavefront slope that can be reliably measured. The dynamic range of the S-H sensor is strictly related to the optical parameters of the S-H microlenses: the lenslet spacing (or number of lenslet across the pupil) and the focal length of the lenslet array. Several methods have been used to increase the dynamic range of the S-H sensor, including a precompensation of LOA, the use of a larger lenslet diameter and/or a shorter focal length lenslet, restriction of the illuminating beam diameter, the magnification of the pupil at the lenslet array *etc.*[25,28–32].

Due to the increasing interest and application of wavefront sensing techniques in the ophthalmic community in recent years, innovative methodologies have been designed and developed. Curvature sensing, pyramid sensing and interferometry are the most promising emerging wavefront sensing techniques.

Curvature sensing technology is based on phase-diversity. It depends on comparisons between phases in adjacent areas in the image and objective plane of an optical system [33–37]. Phase diversity is normally implemented by recording two images at different focal planes, and then reconstructing the wavefront from these images. Aberrations in the wavefront in the measurement plane will alter the local intensities of the wavefront as it propagates. A convex distortion will cause the wavefront to converge and hence become more intense, the contrary will be the case for a concave distortion. The change in intensity is a measure of the local wavefront curvature and may be used to reconstruct the wavefront. The Curvature Sensor (C-S) was originally developed for astronomical observation based on the technique developed by Roddier in 1988 [38]. His original idea was to directly couple a curvature sensor element and a bimorph deformable mirror, without the need for intermediate calculations, in an AO astronomical telescope. Experimental work by Diaz-Douton *et al.*[39] has demonstrated the feasibility of a curvature sensor for ocular wavefront measurement. The advantages of the C-S are its relatively high dynamic range and low cost, while the disadvantages are related to the prolonged time of computing and the fact that a large defocus is needed to measure the wavefront with higher resolution, thus reducing the sensitivity of the sensor. This means that the C-S might not be as accurate as expected for measuring HOA. On the other hand, this problem could be overcome by designing a C-S in which the defocusing distance can be adjusted to the individual eye [25].

The pyramid sensor (P-S) was developed by Ragazzoni and implemented for the first time in an astronomical AO system [40,41]. In this system, a transparent pyramid splits the stellar image into four parts. Each beam forms an image of the telescope pupil on the same detector. A four-faceted refractive pyramid is placed in the optical path with its apex aligned to the optical axis facing the incoming beam. The wavefront gradients along two orthogonal directions are retrieved from the intensity distribution among the four pupil images resulting from this operation. The first application of a P-S sensor in ophthalmology was demonstrated by Iglesias *et al.*[42] in 2002, who used this method to measure the WA of artificial and normal eyes. They also pointed out the necessity to deal with spurious reflections from the anterior cornea. Chamot *et al.*[43] developed an ophthalmic AO system with a P-S in the measurement arm of the device and a piezoelectric deformable mirror (DM) as wavefront corrector element. The DM was optically conjugated to a steering mirror positioned in the wavefront sensor arm immediately before the tip of the diffractive pyramid element; this modulates the position of the image on the pyramid tip in a circular manner and facilitates quantitative measurement of the wavefront slopes. Tests were performed in artificial and human eyes achieving results similar to those obtained by other AO systems implemented with a S-H sensor, further demonstrating the feasibility and accuracy of the P-S sensing technique in an AO system for ophthalmic applications. One of the main advantages of a P-S is the easy adaptability of the system to the variations in the range of the aberrations one can expect in the human eye optics. The dynamic range of the sensor can be therefore quickly modified. Moreover, at small modulation amplitudes the sensitivity of a pyramidal sensor can be higher than that of a S-H sensor.

A variety of interferometry techniques have been suggested for ocular wavefront sensing, including shearing interferometry [44–49] and Talbot interferometry [50–52]. The common advantages of all the interferometric techniques is their relatively simple and inexpensive design if compared to other opto-electronic systems, as well as their accuracy, high spatial resolution and large dynamic range. The disadvantages are the sensitivity to vibration, changes in polarization of the beam coming back out of the eye and the complex reconstruction of the phase error. All these factors limit widespread application of this technology to human eyes. Among the interferometric techniques, Talbot interferometry has gained the most interest for application in vision science [25]. It is constructed with two gratings in which moiré fringes are generated by superimposing the Fourier image of the first grating on the second. The two gratings should have the same period. If the phase object is placed in front of the first grating, the light deflected by the object yields the shifted Fourier images and the resultant moiré fringes show the deflection mapping [50]. On the other hand, only one periodic grating can be used for phase distortion analysis, by exploiting the Talbot effect or self-imaging phenomenon [52]. The Talbot image can be directly detected by a detector placed at the Talbot distance from the periodic pattern, as described in previous work [25]. Distortion of the fringe pattern reflects the local tilt of the wavefront. This pattern can be observed only at a very short distance from the grating due to diffraction and the pattern disappears as distance increases. Diffraction patterns can be observed again at specific periodic distances from the grating. These are called Talbot images. Sekine *et al.*[53] used a two-dimensional grating for sensing the optical wavefront with the CCD placed in the plane of the Talbot image of the first order to maximize the contrast of the grating image. They obtained Talbot images from both artificial and human eyes and were able to successfully reconstruct wavefront shapes, with no discernible differences in comparison with those obtained by a S-H sensor. Warden *et al.*[54] demonstrated accurate results using a Talbot wavefront sensor which has recently become available commercially. A series of measurements was taken in model eyes demonstrating the high accuracy of the Talbot sensor in comparison with two commercially available S-H sensors, especially for HOA.

## 5. Wavefront Correcting Technology

Wavefront correction has been performed by different techniques, depending on the source of aberrations, the field of application, and the instrumentation available. The correctors fall within two broad categories: (1) piston segmented devices [e.g., piston-tip-tilt, liquid crystal spatial light modulators (LC-SLM) and segmented mirrors] and (2) continuous surface mirrors (e.g., including deformable mirrors, membrane micro-mirrors, and bimorphs). The correction methods range from phase conjugation and computer-generated holograms to deformable mirrors, which nowadays are the most popular devices for adaptive optics.

Wavefront phase errors can be corrected by introducing an optical path difference in the beam by either varying the refractive index of the phase corrector (refractive devices) or by introducing a variable geometrical path difference (reflective devices *i.e.*, deformable mirrors). The most common refractive devices use liquid crystals, the refractive index of which can be electrically controlled. Liquid crystal (LC) molecules are elongated and are polarised such that intermolecular forces between the crystals keep them relatively aligned. The basic form of a LC device consists of a layer of LC sandwiched between two pieces of optical quality glass. The orientation of the LCs is fixed by cutting microscopic grooves on the inner surfaces of the glass plates; the LCs line up in the direction of the grooves. Transparent electrodes are deposited on the glass surfaces, and they may be as small as 10 μm. LC devices can therefore provide many thousands of correction elements. These devices offer piston-like correction since there is practically no continuity requirement for the refractive index between pixels. They come in different varieties: (1) ferroelectric devices can produce phase changes of either 0 or pi radians and can operate at high frequencies; (2) nematic devices can provide continuous phase changes but are slower than ferroelectric devices. The first use of LC displays (LCDs) for dynamic ocular wavefront correction was by Prieto *et al.*[55]. More recently, ocular wavefront correction has been demonstrated using Liquid Crystal on Silicon devices (LCoS) [56]. In this device the LC is deposited on an array of silicon pixels and operated in reflective mode. The voltage applied to the pixels controls the refractive index, and the advantage is that higher speeds can be obtained. LC devices require the use of linearly polarised light [56]; Kong *et al.*[57], however, recently described an open loop ophthalmic AO system in which the uncorrected polarisation component is used by the wavefront sensor. A limitation of LC devices is the relatively limited dynamic range when using the device to correct ocular aberrations.

Deformable mirrors (DM) consist of a reflective faceplate acted on by a set of actuators. The faceplate can be continuous or segmented. While it is easier to manufacture segmented mirrors with a very large number of actuators, they have the disadvantage that the gaps between segments introduce greater spurious effects in the image (due to diffraction) than continuous mirrors. Many different types of actuators have been used and most of them have been applied to ocular AO systems [58]. The most common types of actuators are piezoelectric, electrostatic or magnetic.

In piezoelectric materials, an applied electric field gives rise to a change in shape. The most commonly used material is lead zirconate titanate (PZT). These mirrors were originally developed for use in astronomical AO [59] and were used in the first ophthalmic AO system [20]. They can be made to feature quite a large stroke, a linear actuation-to-voltage response, and low amounts of hysteresis. The typical size of a PZT actuator is 25 mm, so deformable mirrors using these actuators tend to be relatively large. Bimorph mirrors consist of two bonded piezoelectric ceramic wafers that are oppositely polarised parallel to their axis [60]. An array of electrodes is deposited between the wafers; applying a voltage to an electrode results in one wafer expanding locally and laterally while the other wafer contracts inducing a spherical bending. These mirrors are a natural choice for AO systems using a curvature wavefront sensor. However they can also be used with Shack-Hartmann (or any other) wavefront sensors, and were used in this way in an ocular AO system [61].

Electrostatic actuators are usually used in membrane-type mirrors or micromirrors [62]. Membrane mirrors consist of a reflective membrane which is deformed by means of electrostatic forces due to an array of electrodes placed a small distance behind the membrane. The local membrane curvature is proportional to the square of the signal voltage. The influence function of the actuators, *i.e.*, the mirror shape when a single actuator has a voltage applied, is broader than those of other mirrors. They are very suitable for low-order correction, and the introduction of commercial models made low-cost adaptive optics possible for the first time. The stroke is limited by the fact that the actuators operate in ‘pull-only’ fashion in the usual configuration. Bonora and Poletto [63] introduced a ‘push-pull’ version having a transparent electrode in front of the mirror in order to extend the stroke. In another interesting development, Bonora *et al.*[64] proposed a “photocontrolled deformable mirror” (PCDM) in which a LCD is used to generate a light distribution on a photoconductive layer placed behind a membrane mirror. The control is therefore by light, and can be very high order or reduced to the order required. Electrostatic actuators are also applied in micromirrors (MEMS). In MEMS the electrodes do not act directly on the membrane, but rather on an intermediate membrane [65]. The mirror is attached to this membrane through posts, and the result is to localise the actuator influence functions. This can therefore provide higher order correction. Currently, there are MEMS by Boston Micromachines (Cambridge, MA, USA) with 32, 140 and 1020 actuators and the actuator size ranges from 300 to 500 μm and the corresponding stroke is from 1.5 to 5.5 μm or by IRIS AO, Inc. (Berkeley, CA, USA), with 111 to 489 actuators with inscribed apertures from 3.5 to 7.7 mm respectively and stroke from 5 to 8 μm.

Magnetic mirrors usually use voice coils to act on small magnets attached to the rear of a membrane mirror. These devices are therefore controlled by current rather than voltage. The main advantage is that a large stroke can be achieved, much larger than for other devices. A 52-Element device (Imagine Eyes, Orsay, France) was demonstrated in an ophthalmic AO system by Fernandez *et al.*[66] and more recently by Lombardo *et al.*[67]. The temporal response of this kind of mirror can be an issue, but it can be taken into account in the control system [68]. The ALPAO company (Montbonnot St. Martin, France) manufactures different versions of magnetic mirror having 37 to 277 actuators with actuator spacing of 1.5 or 2.5 mm and overall stroke up to 60 μm. They claim settling times of order 1 ms for their “hi-speed” range.

A novel magnetic fluid mirror is receiving attention [69]. In this device the surface of a magnetic fluid is coated with a reflective film and acted on by coils placed under the fluid volume. The deformation depends on the square of the magnetic field, but can be linearised by the addition of a strong, uniform magnetic field. The stroke can be large, and the response can be made fast by using high-viscosity ferro-fluids. However, these liquid mirrors must conserve volume which means that superposition of influence functions is not possible. This and the fact that they can evaporate are the main disadvantages of liquid mirrors. A liquid mirror which is electrostatically deformed has also been demonstrated recently [70].

The performance of wavefront correctors depends on a number of parameters, including the number and configuration of the actuators, the stroke, temporal response, linearity, hysteresis *etc.* Devaney *et al.*[71] compared eight different commercially available mirrors for correcting both ocular and atmospheric wave aberration. The sample included piezoelectric, membrane, bimorph and magnetic mirrors. Influence functions of all the mirrors were measured using interferometry. Wavefronts were simulated to have statistics corresponding to either ocular or atmospheric aberrations, with the correction achieved for each mirror determined by least-squares fitting the influence functions of each mirror to the wavefront. The number of mirror modes corrected and the size of the optical pupil projected on the actuator geometry were optimized for each device. In general, it was found that better correction can be obtained when there is a ring of actuators just outside the pupil. The optimal number of modes to correct depends on the mirror stroke and geometry. It was found that the mirrors with higher stroke (the magnetic and bimorph devices) should provide the best performance in terms of residual root-mean-square (RMS) wavefront error or Strehl ratio.

## 6. Applications of Adaptive Optics Retinal Imaging

**Figure 3.**
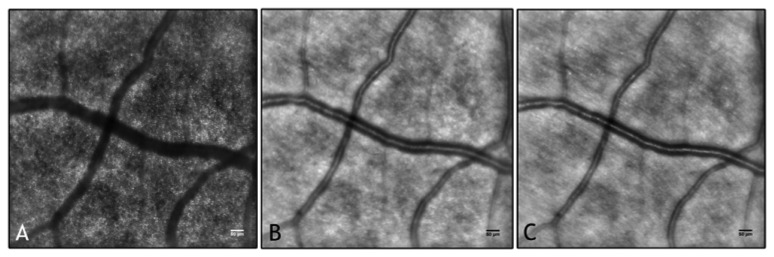
AO retinal images with adaptive compensation of the photoreceptor layer (**A**), vasculature (**B**) and retinal nerve fiber layer (**C**). Scale bars represent 50 μm. The blood vessels form a three-dimensional network across the inner retinal layers. All the images shown in this review have been acquired using a flood-illumination AO retinal camera (rtx1, Imagine Eyes, Orsay, France).

Adaptive optics retinal imaging has demonstrated the capability to image the living human retina at microscopic resolution. The improvement in retinal image contrast and resolution allows the direct observation of retinal microstructures giving the researcher the opportunity to analyze their integrity and/or pathological abnormalities. An AO ophthalmoscope provides en face images of the retinal layers showing photoreceptors, retinal vessels and nerve fiber bundles (Figure 3). Advances in image processing methods are required in order to maximize the value of the retinal data acquired and to make the AO retinal imaging technology accessible to the clinical ophthalmic community. Accurate automated routines are indeed mandatory when large quantities of data need to be analyzed.

**Figure 4.**
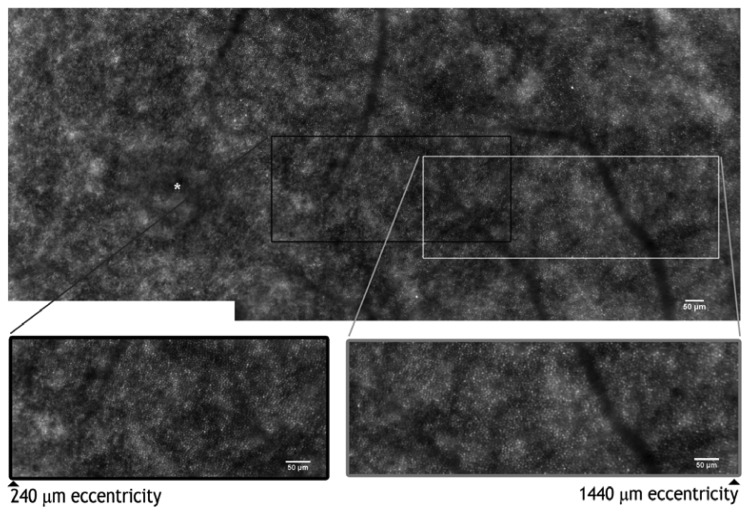
AO montage of the photoreceptor mosaic in a 46 year old subject. The asterisk shows the foveal center. The black and grey boxes enclose two high-magnification images of the photoreceptor layer from 240–1,440 μm eccentricities from the foveal center. The center-to-center distance between cones has been shown to increase with greater eccentricities from the fovea; the cone density, accordingly, declines with increasing eccentricity from the fovea. Scale bars represent 50 μm.

Currently, the majority of studies have focused on the generation and analysis of AO images of photoreceptor cells, including cones and rods [67,72–75]. Over the last few years, efforts have been made to develop reliable methods to measure the cone density as a function of retinal eccentricity in populations of healthy adults [76–81]. In general, data on populations of healthy eyes are fundamental for characterizing the density and the spacing distribution as well as the brightness of healthy photoreceptor cells *in vivo* (Figure 4). This will allow measurement of the normal ranges which can be compared to pathological photoreceptors, even in the early stages of retinal diseases. An increasing number of studies [67,82–90] are showing the distribution of cone photoreceptor density and spacing in adults. The *in vivo* measurements of cone density [67,82–85] have shown good agreement with histologic data from cadaver eyes [86–90]. Using an AO scanning laser ophthalmoscope (AOSLO), researchers [82–85] found the cone density to drop on average from 120,000 cones/mm^2^ at 0.1 mm to 20,000 cones/mm^2^ at 1.0 mm from the foveal center. In general, the cone density values at the same eccentricities of the nasal and temporal retina within 2 mm eccentricity from the fovea were found to be within 10% of each other both in *ex vivo* and *in vivo* studies [84–87,91]; a 10% higher density of cones along the horizontal than the vertical meridian was, in general, found. A synopsis of the average cone density from previous AO studies in populations of healthy subjects is shown in Table 1. The parafoveal cone density showed a moderate to high inter-individual variation with coefficient of variation (defined as the ratio of the standard deviation to the mean) values ranging between 12% and 20% [82–86,91]. Discrepancies between studies could be due to different factors, such as the inclusion of subjects with different ages or eyes with different axial lengths and refractive corrections, the instrument used for biometry, the different model eye used to estimate the retinal image size, the use of foveal center or foveal fixation as reference point to define retinal eccentricities and the sampling window area used to count cones.

AO retinal imaging in healthy subjects also makes it possible to better understand the sampling limit of resolution of the cone mosaic *in vivo*. Analysis of the spatial distribution of the cone photoreceptors provides new information on the physical aspects of visual sampling of the human eye. In a recent work, our group [91] found that the mean Nyquist limit sampling of resolution of the cone mosaic (N_c_) was 33 ± 2 cycles/degree (c/deg) at 260 μm eccentricity, declining to 26 ± 2 c/deg at 600 μm eccentricity in a population of twelve young adults (age range: 24–38 years; 24 eyes; axial length of the eye (AxL) range: 22.61–26.63 mm). Authors previously found comparable results for young adults [83,84,92]. The N_c_ was calculated to be 34 c/deg at 1° eccentricity (approximately 270 μm) by Chui *et al.*[84]. Coletta and Watson [92] estimated N_c_ to range between 50 and 42 c/deg at the fovea and between 24 to 22 c/deg at 4° eccentricity (approximately 1.1 mm) in a population of subjects with spherical equivalent error (SEr) ranging between 0 D and −14 D. In a population of 18 healthy subjects (age range: 23–43 years; 18 eyes: AxL 22.86–28.31 mm), Li *et al.*[83] found that cone density tended to decrease with increasing axial length at eccentricities between 100 and 300 μm from the foveal center, with no statistically significantly differences between emmetropes and myopes at the fovea. In a population of 11 healthy subjects (age range 21–31 years; 11 eyes), cone density in moderate myopes (up to−7.50 D) was found to be significantly lower than in emmetropes within 2.00 mm eccentricity from the fovea [84]. The spatial vision of the cone mosaic reduces with increasing axial length [91]: the lower Nyquist limit sampling of resolution of the cone mosaic in myopes than emmetropes has been postulated to be caused by retinal stretching, occurring at the posterior pole of myopic eyes, due to the eye's increased axial length [84,91,92]. A higher difference between myopes and emmetropes has been demonstrated when the acuity limits were expressed in retinal units (c/mm) rather than in angular units (c/deg). The discrepancy between resolution and N_c_ expressed in retinal acuity was primarily considered a result of psychophysical and neural factors, including the neural sampling rate (*i.e.*, the retinal ganglion cells receptive field density) [93–95].

AO retinal imaging has also been used to study the waveguide and reflectance properties of cone photoreceptors *in vivo*[96–104]. Variation of brightness between adjacent cones was observed, even when imaging the retina of healthy subjects. Differences in reflectance between adjacent cones were seen both at the boundaries of retinal vessels and in areas devoid of vessels. While intra-retinal scattering was considered as the primary source of the higher reflectance of cones that reside beneath the vessels in comparison with adjacent cones, this phenomenon could not explain the variation in brightness between adjacent cones in areas devoid of vessels. Various hypotheses have been made to find the possible factors that influence the intensity variation of the cone mosaic: (1) the differences in reflectivity could be caused by molecular differences within the cones that are due to phototransduction [96]; (2) the reflectance variation could be based on the cone outer segment length [97–100,102–104]: fluctuations in reflectivity indeed could be due to changes in the outer segment length, related to disk shedding. (3) The cause of the variation could also be related to the pointing direction of cones and the angle of incidence of the light entering the pupil [105–108]. (4) Finally, technical factors should be considered to contribute to differences in reflectance between adjacent areas of cones, including the light source (*i.e.*, laser or SLED emitter) and/or the wavelength of light used to illuminate the retina, the axial resolution of the imaging system (confocal or flood-illumination) and the imaging process [100,101,104]. The relative contribution of the sources of reflection within the cones was shown to be not constant with time [98,100,102,103] and the photopigment density has been found to decrease with increasing eccentricity from the fovea up to 2° eccentricity [109].

## 7. Adaptive Optics Retinal Imaging of Retinal Diseases

In addition to the above basic applications, ophthalmic AO systems are translating into clinical applications that are rapidly expanding. It is expected that AO technology will soon translate into clinical applications. The most promising application of AO retinal imaging is the detection of early signs of retinal diseases. AO retinal imaging indeed provided new information about the pathological changes of the retinal microstructure in several diseases, including inherited or acquired neuro-retinal degenerations [110–112] and vaso-occlusive diseases [113,114]. Because the rate of disease progression is typically slow for most retinal degenerations, it is estimated that patients show a clinical loss of visual function years after the onset of the disease. If AO imaging can provide high-resolution measurements of the cone photoreceptor structure, micro-vasculature and nerve fiber bundles in patients, then it may open a new frontier for the early diagnosis of retinal diseases and for monitoring the efficacy of therapies at a cellular level. Researchers [7,85,110] have demonstrated that AO devices allow imaging of exactly the same retinal area over days or months in order to follow disease progression with microscopic accuracy.

Across the developed world, the major causes of vision loss are attributed to diabetic retinopathy (DR), age-related macular degeneration (AMD) and glaucoma [5]. Diagnosis usually occurs once the damage has already happened to some extent, because of the relatively low resolution of current retinal imaging techniques when used to detect abnormalities of the retinal microstructures and the relatively poor sensitivity of functional testing. Considering that structural damage of these microstructures precedes their functional impairment, the detection of pathological variations of photoreceptors, capillaries and nerve fiber bundles at the pre-clinical stage of the disease can be beneficial for early treatment and avoidance of serious visual loss. Furthermore, a better understanding of the microscopic alterations in retinal tissue may provide further insight into the mechanisms of disease progression and be helpful to identify new approaches for therapeutic intervention.

Diabetic retinopathy (DR) is a frequently occurring complication of diabetes mellitus, that is a metabolic disease in which the patient has a high serum glucose level. According to the World Health Organization, diabetes mellitus is responsible for about 12% of new cases of blindness between the ages of 45 and 74 years in the developed world. With the incidence of diabetes throughout the world projected to rise from 150 million to approximately 300 million by the year 2025, DR represents a major threat to the global population and will likely present ever-increasing burdens on the health care delivery system [115,116]. DR can be classified as non proliferative (NPDR) or proliferative (PDR). NPDR is further graded as mild, moderate and severe according to the Early Treatment Diabetic Retinopathy Study (ETDRS) severity scale [117].

The earliest clinical pathological changes of DR occur in the microvascular structures [118,119]. According to the most commonly accepted patho-physiological model (*i.e.*, the microvascular theory) [120], DR consists of a microangiopathy that induces pathological changes of the vascular structures and the blood rheological properties as a consequence of chronic hyperglycaemia. It has been also postulated that DR is a multifactorial disease involving the retinal neuronal cells (neurodegenerative theory) [121–124]. The neurodegenerative changes are apoptosis of several populations of retinal cells, including photoreceptors, bipolar and ganglion cells. The functional and structural impairment of neural tissue has been theorized to participate in the generation of the earliest morphological alterations of the vascular structures [121–125]. The question of whether the photoreceptor loss could be initially determined by a neuronal or vascular breakdown during diabetes mellitus remains unsolved. Work is needed to understand whether the neurodegenerative changes of retinal cells may precede the vascular damages or the two processes are simultaneous. The exact nature of their interdependence is complex and still not known.

**Figure 5.**
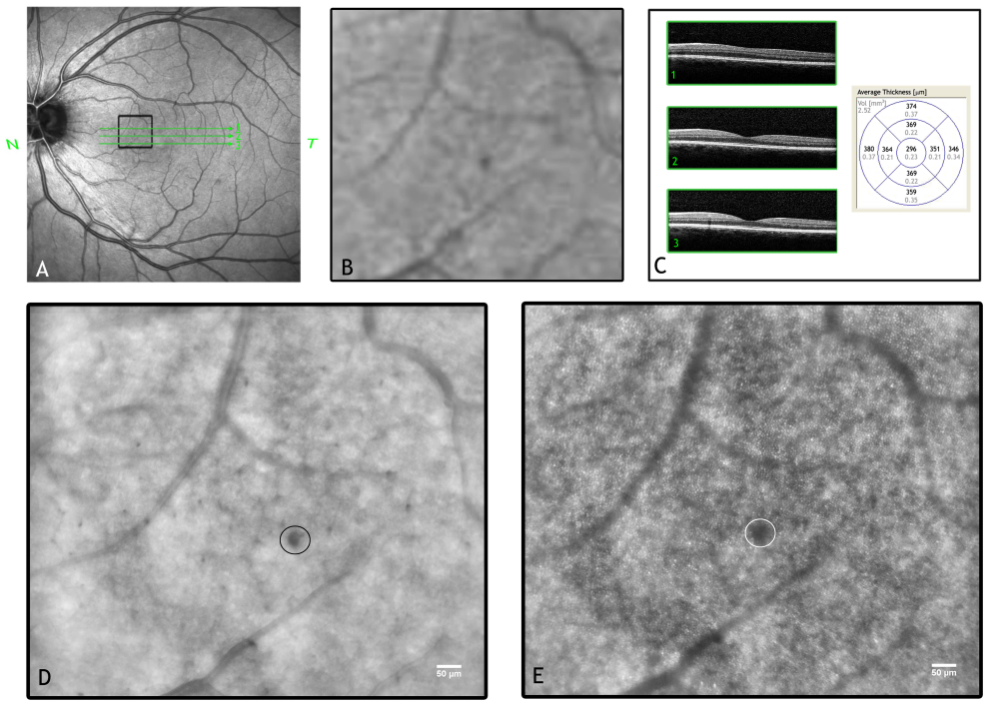
(**A**) Wide-field SLO image of the left eye in a 38-year old female patient with type 1 diabetes and mild non proliferative diabetic retinopathy. (**B**) High-magnification SLO image of a region of interest (encircled in A, 0.88 × 1.53 mm) showing a micro-haemorrhage, though with low resolution. (**C**) The SD-OCT horizontal scans of the central retina, corresponding to the green lines superimposed to the SLO image in A show a preserved retinal microstructure. The retinal thickness map is also shown. (**D** and **E**) The same region of interest imaged by an AO system with adaptive compensation of the vessels and photoreceptor layer, respectively. Scale bars represent 50 μm. In (**D**), the small arterioles and the capillaries create a web between larger vessels; the spot haemorrhage (encircled) shows distinct margins. In (**E**), the AO image of the cone mosaic. The haemorrhage projects a dense shadow, with defocused margins, onto the mosaic completely masking the underlying photoreceptors.

**Figure 6.**
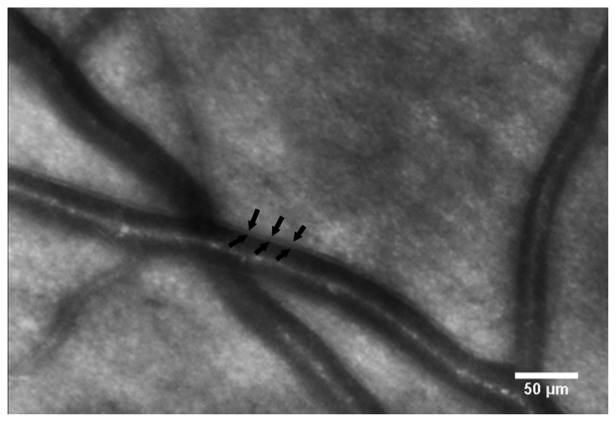
AO image showing a retinal artery. In en face retinal imaging, the lumen of a blood vessel appears as a streak of variable diameter and morphology, depending on the vessel order; however, it always shows the same pattern, consisting of a central high-intensity channel and two peripheral darker channels (likely due to the curved vessel wall). The artery wall appears as a grey line outside the peripheral lumen vessel. The arrows define the inner and outer wall borders. Scale bar: 50 μm.

Noninvasive detection of the pathological signs of DR is usually performed by dilated fundus examination, colour fundus photography and more sophisticated retinal imaging techniques, such as Spectral Domain Optical Coherence Tomography (SD-OCT). Fluorescein angiography (FA) can be useful to assess the integrity of the blood retinal barrier as the amount of fluorescein leakage is related to the dysfunction of the retinal vascular endothelium. Even if FA represents an important diagnostic tool and improves the accuracy of laser treatment of DR complications (e.g., diabetic macular oedema and new vitreo-retinal vessels) [126,127], on the other hand, it requires injection of a fluorescent dye agent that can lead to unintended systemic complications. AO retinal imaging could be useful to detect pathological changes early in the course of DR, such as microaneurysms, micro-haemorrhages and loss of photoreceptors [113,114,128–130] (Figure 5). The detection of the early retinal changes related to DR might be important in the management of the patient requiring a better glyco-metabolic control. Authors [128,129] described image processing and analysis algorithms to extract the capillary vessel information and provided excellent visualization of the parafoveal capillary network in healthy eyes using AOSLO devices. In a recent study [114], the capillary network was evaluated in patients with type 2 diabetes. Researchers found a higher capillary dropout and a higher tortuosity of the arterovenous channels in patients with diabetes and no diabetic retinopathy than in healthy controls. Tam *et al.*[113] analyzed the retinal microvasculature in a patient with type 1 diabetes and severe NPDR over a 16 months follow-up period. Longitudinal assessment of the capillaries showed microaneurysm formation and disappearance as well as the formation of tiny capillary bends similar in appearance to intraretinal microvascular abnormalities. *In vivo* imaging of the capillary network has been also shown using an AO-SD-OCT [131–133]. The 3D information provided by OCT represents a major advantage compared to *en face* imaging techniques. The AO-SD-OCT can provide a theoretical spot volume of 3 μm^3^, capable of reconstructing the entire retinal capillary network of the inner retina. On the other hand, OCT cannot distinguish the lumen from the vascular walls of larger vessels [134]. In a preliminary investigation on large retinal arterioles in patients with type 1 diabetes and NPDR, we acquired images of the vessel walls using an AO flood-illumination retinal camera (Figure 6 and Movie 1 (movie showing a retinal artery, as seen in Figure 6). The blood vessel shows periodic twitching, probably corresponding to the cardiac cycle of the patient, as previously shown by Zhong *et al.*[135], showing the capability of AO ophthalmoscopy to evaluate them for monitoring both arterial lumen and walls in vaso-occlusive retinal diseases. Both axial and en face AO imaging techniques could therefore be complementary to noninvasively analyze the retinal vessels at high resolution. The detection of pre-clinical abnormalities of retinal microcirculation in patients with diabetes could represent a valuable advantage of AO retinal imaging in comparison with current noninvasive imaging modalities.

**Figure 7.**
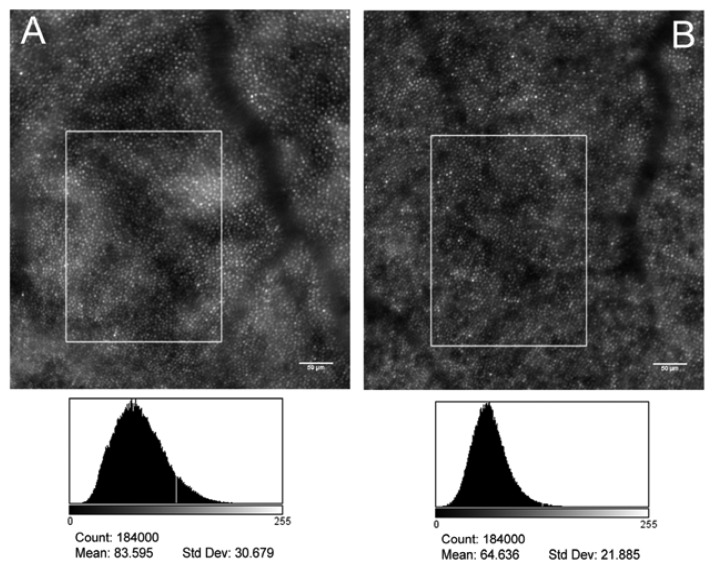
AO images, both centered at coordinates: x = 1.5° temporal and y = 1.8° superior from the fovea, of the photoreceptor mosaic in the left eye of a patient with type 1 diabetes without clinical signs of diabetic retinopathy (**A**, 36 years old female, noDR eye) and a healthy control (**B**, 37 years old female). Scale bars represent 50 μm. In the lower row, the image histograms (x-axis: 0–255 grey level; y-axis: pixel intensity level) of the selected areas are shown: both a higher average and a higher standard variation of the intensity level distribution of pixels was found in the noDR eye than in control. Biological and technical factors can contribute to variations in brightness between adjacent domains of photoreceptors.

**Figure 8.**
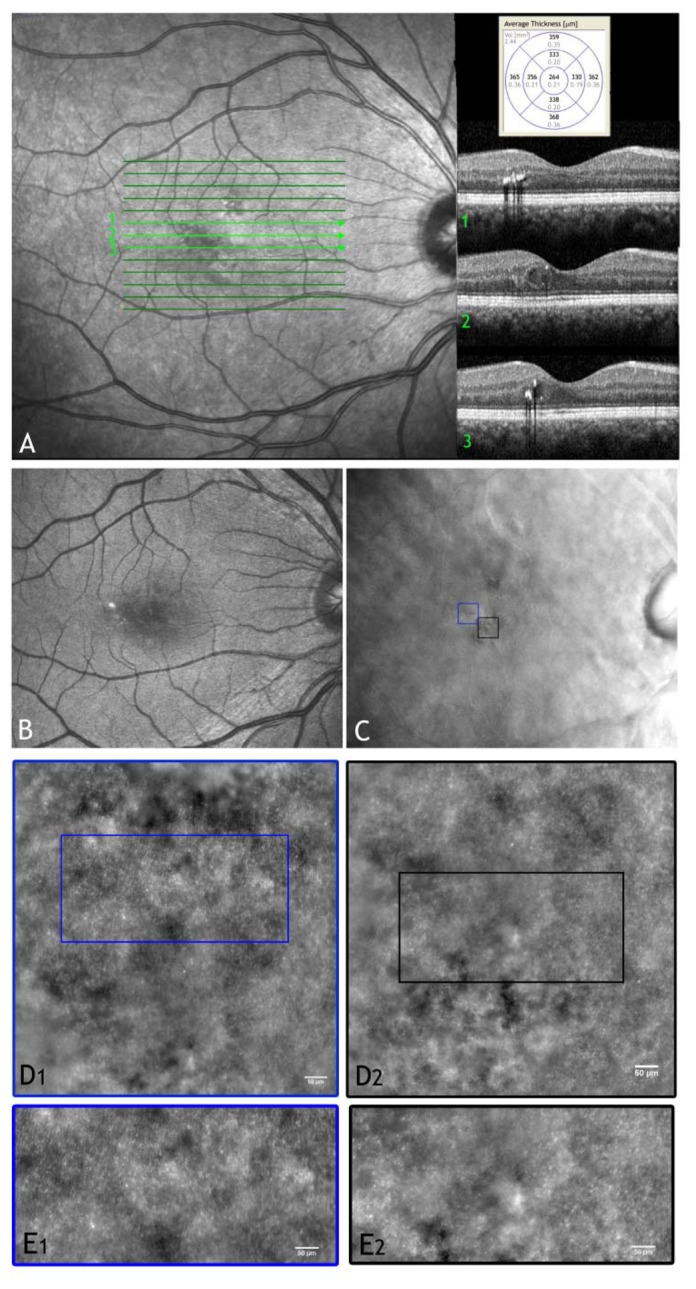
(**A**) Wide-field SLO image and OCT scans of the right eye in a 49 year old patient with mild non proliferative diabetic retinopathy showing hard exudates and a focal macular oedema. (**B** and **C**) Wide-field digital images (retromode modality by F10, Nidek, Japan) of the posterior pole showing the locations of the retinal exudate and the micro-cystic oedema (black box), respectively. (**D1** and **D2**) Adaptive optics images of the photoreceptor layer acquired within the regions of interest enclosed in C (scale bars represent 50 μm). In panel D2, cones are highly resolved only in part probably due to increased scattering from oedematous inner retinal layers. (**E1** and **E2**) High-magnification images of the photoreceptor layer shown in D1 and D2 respectively (scale bars: 50 μm). High variations in brightness between adjacent domains of photoreceptors can be seen in regions close to retinal oedema (E1). Intraretinal oedema reduces high-resolution imaging of photoreceptors (E2).

Non-necrotic photoreceptor loss, in addition to microangiopathy, has been considered to be responsible for the vision loss associated to DR [121–124]; on the other hand, a loss of cone photoreceptors has never been clinically demonstrated. An objective of our current work is to evaluate the photoreceptor mosaic geometry and reflectance: the procedure has the potential to provide additional and valuable information about the cellular changes of retinal pathologies. In a recent study [136] we found that, in a population of eleven patients with type 1 diabetes, the parafoveal cone photoreceptors showed a higher variation in intensity than in healthy controls at the same retinal eccentricity (Figure 7). This phenomenon was particularly evident near the areas of intraretinal focal oedema (Figure 8). The regional differences in image intensity with areas of cones brighter than adjacent areas were also previously seen in other retinal diseases, including cone-rod dystrophy [110]. The significance of this phenomenon is not yet clear. In general, there are various hypotheses on the intensity variation of cones in AO imaging, as discussed in the previous section [97–104]. Fluctuations in reflectivity may be caused by molecular changes within the cones that are due to phototransduction or to changes in outer segment length, related to disk shedding. It has been also theorized that areas of cones darker than adjacent cones may reflect a disruption of the waveguide properties of the cones themselves [105,109] or to light interactions between the end of the OS tip and the pigments in macrophages or retinal pigment epithelial (RPE) cells [102,103]. Technical factors, however, should be considered to contribute to the spatial variation of reflectance between adjacent areas of cones, as previously discussed [100,102,104].

Age Related Macular Degeneration (AMD) is the leading cause of blindness in the elderly across the developed world. It represents a deterioration of the macular area and usually affects older adults. A recent study found that the late stage AMD (the most disabling form of the pathology) is present in approximately 5% of the over 65 's and 12% of the over 80 's [137]. AMD is a multifactorial disease, involving ocular, systemic and genetic risk factors. The ocular risk factors include darker iris pigmentation and hyperopic refraction, while systemic risk factors include cigarette smoking, obesity, sunlight exposure and cardiovascular diseases [138,139]. Genes influence several biological pathways related to AMD, including the immune processes, mechanisms involving collagen and glycosaminoglycans synthesis and angiogenesis. All these factors have been associated with the onset, progression and bilateral involvement of early, intermediate, and advanced states of AMD [140–146]. Genetic susceptibility can be influenced by the environmental factors: taken together, both factors are highly predictive of the onset, progression and response to treatments [147]. Several patho-biological pathways have been implicated in the pathogenesis of AMD: these include senescence, shown by a lipofuscin accumulation in the RPE cells, choroidal ischemia and oxidative damage [148,149].

There are two clinical types of AMD, the “dry” and “wet” form. In the early stages of AMD, which is asymptomatic, insoluble extracellular aggregates called *drusen* accumulate in the retina. The late stage of dry AMD, which is also known as *geographic atrophy* (GA), is characterized by scattered or confluent areas of degeneration of RPE cells and the overlying retinal photoreceptors, which rely on the RPE for trophic support. The other late stage form of AMD, the wet form (10–15%), is typified by *choroidal neo-vascularization* (CNV), where newly immature blood vessels grow toward the outer retina from the underlying choroid leaking fluid below or within the retina [150,151].

Retinal imaging for the management of AMD includes fluorescein angiography. In the wet AMD form, leakage of dye (hyperfluorescence) is noted and classified by location (subfoveal, juxtafoveal, or extrafoveal) and by type (classic, occult, or mixed). Indocyanine green angiography (ICG) uses an intravenous dye with different characteristics from fluorescein (e.g., less melanin absorbance). It improves identification and characterisation of neovascular variants of AMD (e.g., the polypoidal choroidal vasculopathy). SD-OCT enables high-resolution *in vivo* cross-sectional or volumetric tomographic visualisation of the retinal micro-architecture. It allows visualisation of the cross-sectional outline of the neovascular choroidal complex, but its internal structure cannot be well resolved and the neovascular components cannot be distinguished from the fibrous components, haemorrhages or dense exudates within the lesion. With the advent of the anti-VEGF therapy, SD-OCT imaging is widely used for the early diagnosis of CNV and for the treatment and re-treatment management.

**Figure 9.**
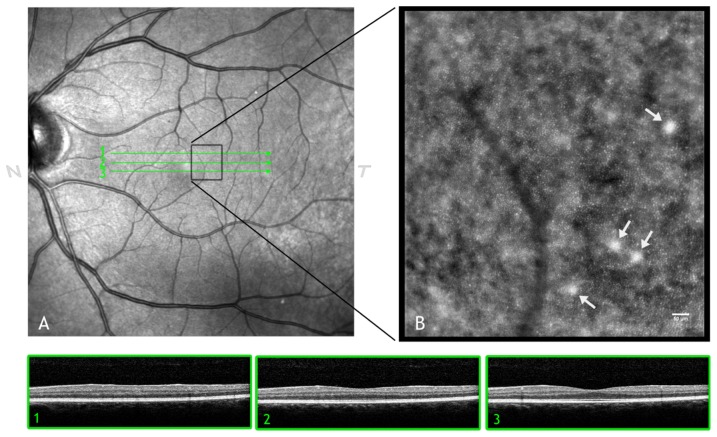
(**A**) Wide-field SLO image of the left eye in a 49 year old male showing hard drusen located near the fovea. The SD-OCT horizontal scans (1, 2 and 3) of both the photoreceptor and retinal pigment epithelial (RPE) layers have almost normal appearances. (**B**) AO image of the region of interest showing *drusen* (white arrows). The cone photoreceptors above drusen are well resolved, showing a higher brightness than adjacent cones. Similar findings have been shown using AOSLO (References [152–154]). Scale bar: 50 μm.

In the early, asymptomatic, AMD stages (*i.e.*, presence of drusen), the ability to predict the rate of progression is currently limited. By monitoring drusen over time, *en face* AO imaging (Figure 9), also combined with AO-SD-OCT imaging, can theoretically detect both their progression, in terms of size, and analyze their direct effect on the overlying photoreceptor mosaic [152–154]. Godara *et al.*[152] showed, for a 45-year old female, that both the cone density and cone arrangement of the mosaic overlying the drusen were within normal limits. AO imaging revealed a regular photoreceptor mosaic with areas of hyper-reflectivity coinciding with the location of the drusen. The increased reflectivity associated with the drusen could be attributed to increased scatter from the RPE (due to decreased melanin or accumulation of some other waste material), to loss of outer segment pigment or loss of the photoreceptor outer segment. Boretsky *et al.*[154] identified several small drusen deposits that were not observed with standard wide field imaging techniques in early AMD. They also investigated large coalescent drusen and areas of geographic atrophy in advanced stage dry AMD, showing significant decrease in visible photoreceptor density. A sensitive, non-invasive, imaging tool could help to better recognize the earliest retinal changes and to identify patients who could progress rapidly and may benefit from a more intensive observation and management. Furthermore in the near future, an early diagnosis of the macular disease could have an important role in the evaluation of the effectiveness of new prevention strategies.

Glaucoma is the leading cause of irreversible, preventable blindness worldwide [155]. It has been estimated that over 11 million glaucoma sufferers worldwide are bilaterally blind from the disease [156]. Primary open angle glaucoma (POAG) is a chronic disease characterized by progressive loss of retinal ganglion cells, usually associated with ocular hypertension, that leads to structural damage of the inner retinal layers, as shown by progressive regional or diffuse thinning of the retinal nerve fiber layer (RNFL) [157]. Axonal tissue loss in the RNFL has been reported to be one of the earliest detectable glaucomatous changes, preceding morphologic changes of the optic nerve head (ONH), followed by functional loss, as shown by progressive visual field (VF) defects. The temporal sequence of glaucomatous structural/functional damage suggests that looking for structural changes at the ONH/RNFL level should theoretically allow an earlier diagnosis than detection of functional defects [158,159].

**Figure 10.**
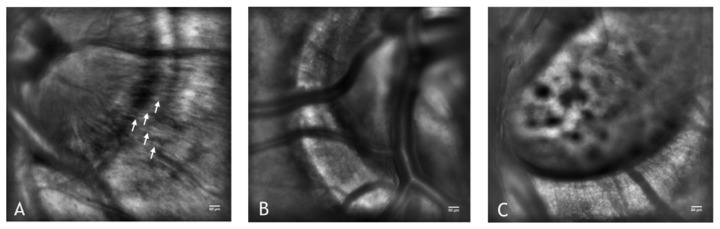
(**A**) The optic nerve head of the left eye in a healthy subject (26 years old female). The nerve fiber bundles can be highly resolved (some are indicated by white arrows) via AO imaging. In (**B**) and (**C**), the optic nerve head in two subjects suffering from advanced glaucoma showing a large papillary excavation. The nerve fiber bundles in advanced glaucoma cannot be resolved as usual in healthy eyes. In C, the lamina cribosa can be seen through the large papillary excavation. A, B and C: scale bars represent 50 μm.

Many imaging modalities have been used to analyze RNFL loss in glaucomatous eyes. Colour and red-free fundus photography [160] represent the standard approaches, but the changes of RNFL are not detectable until there is more than 50% nerve fiber loss. Scanning laser polarimetry, scanning laser ophthalmoscopy and SD-OCT are imaging modalities that allow a quantitative analysis of the ONH and RNFL. Commercially available OCT devices, however, cannot provide sufficiently clear images of individual nerve fiber bundles to identify the specific structural abnormality that underlies the pathogenesis of glaucoma [161,162]. Adding AO to imaging systems such as flood-illuminated ophthalmoscopes, SLO equipment or OCT has recently allowed researchers to identify individual nerve fiber bundles [163–165], providing high-resolution images of both the RNFL and the ONH (Figure 10). Takayama *et al.*[163], using an AOSLO, measured the individual nerve fiber bundles width in a population of twenty healthy adults. In all the eyes, the AOSLO images showed hyperreflective bundles, representing the nerve fiber bundles, in the RNFL. Dark lines among the hyperreflective bundles were considered to represent Müller cell septa. The width of nerve fiber bundles, at distances from the edge of the optic disc ranging between 1.00 and 6.00 mm, was 22 ± 6 μm. There were no significant differences among the bundle widths at these distances along the same meridian. The hyperreflective bundles on the temporal and nasal sides of the optic disc were, however, narrower (on average 20 μm width) than those above and below the optic disc (on average 30 μm width). In the central retina, the hyperreflective bundles nasal to the fovea were narrower than those above or below the fovea.

Kocaoglu *et al.*[165] used an AO-OCT to obtain images of the RFNL in four healthy subjects. The imaging sessions were confined to three locations: retinal eccentricities of 6 degree superior and inferior to the fovea, and 3 degree nasal to the fovea. The authors showed that the nerve fiber bundles reflect noticeably more light than the surrounding tissue, a factor of approximately two times more. As they approach the fovea, the nerve fiber bundles become thin (both in width and depth) and separate. Bundles at 3 degrees demonstrate a larger aspect ratio (width to thickness) than those at 6 degrees with average width and thickness ranging from 30–50 μm and 10–15 μm, respectively.

Currently, there are no studies on glaucomatous eyes or eyes with ocular hypertension. AO imaging could indeed be useful to evaluate the morphological characteristics of the RNFL in patients with ocular hypertension in order to identify possible risk factors implicated in the ONH damage progression. AO could also help to recognize early glaucomatous damages and to identify patients who could progress rapidly and also may benefit from more intensive observation and management. Moreover, AO imaging could have an important role in the evaluation of neuroprotection strategies.

## 8. Conclusions

The optical system of the eye imposes a limit for retinal imaging. If the eye's wavefront aberrations are completely corrected across the pupil, significant improvement in the eye's optical quality is gained. Such improvement has been shown to be valuable in recent clinical applications of adaptive optics retinal imaging [110–114,152,154].

Over the last ten years, advances in understanding of the eye's aberrations have moved the wave aberration theory from an academic concept to an engineering level that is central to improving ophthalmic technologies. In the first experiment using a fundus camera equipped with adaptive optics, Liang *et al.*[20] were able to image individual cones in the living human retina. Since this seminal paper, adaptive optics for retinal imaging applications has entered the field of clinical research. The ability to image the photoreceptor layer, the retinal micro-vasculature and the nerve fiber bundles *in vivo* now provides the opportunity to better understand the pathological processes leading to visual impairment [72–90,129–131,163–170]. The future of AO retinal imaging promises early detection of degenerative retinal diseases and monitoring the efficacy of treatments at a cellular level. In retinal diseases, early detection and treatment are essential to prevent the occurrence of serious damage and visual loss. It has been shown that it is possible to take images, with cellular resolution, in exactly the same retinal area over days, months and years [85,110,171]. The ability to longitudinally track disease progression serves as the foundation for an imaging-based approach to track treatment response with greater sensitivity and on a much shorter time scale than current outcome measures such as visual acuity and visual field sensitivity can allow. As novel treatments to slow disease progression in both inherited and acquired retinal degenerations are developed, it will be critical to evaluate the effect treatments have on individual photoreceptor cells. It is expected that AO high-resolution imaging tools will allow clinicians to track retinal disease and the efficacy of therapy with great accuracy, helping to accelerate the search for new strategies of secondary prevention to avoid serious visual loss.

High cost and system complexity currently hinder the wide adoption of AO technology in clinical ophthalmology. Most AO retinal cameras have been designed and constructed for the best imaging performance possible, with the exclusion of all other factors, including size, cost, complexity, ease-of-use, time required to obtain, process, and analyze the retinal images, *etc.* This is delaying the transition of AO from the research lab to the clinic. Nevertheless, important progress has been made in this regard during recent years [6,172]: with reports of significant performance improvement of AO methods and systems for a growing number of ophthalmic applications, demand for a “commercial” AO instrument will increase and its cost should probably decrease. A compact, simplified AO instrument that can be used by ophthalmologists will facilitate the introduction of this technology into clinical practice and the development of new methodologies to detect and treat retinal diseases. Four companies have developed an AO prototype as a clinical viable tool at the time of this review (Boston Micromachines Corporation; Canon, Inc.; Imagine Eyes; and Physical Sciences, Inc.).

Another current drawback of AO imaging is the time required to obtain, process and analyze the retinal images: the continuous advances in the development of automated and reliable methods to evaluate the retinal micro-structures, including cell photoreceptors, vessels and nerve fiber bundles [82–85,113,114,128,129,131,163–165] is however expected to resolve this issue soon. Accurate automated routines are indeed mandatory when large quantities of data need to be analyzed. A number of research groups are evaluating methods of analysis and interpretation of AO retinal images, such as cone density and spacing and packing regularity of the cone mosaic. Functional features of the cone mosaic can be used to capture additional information that cannot be described by the above metrics, such as the variation of cell brightness between adjacent domains of healthy and abnormal cones [153]. Continuing efforts to develop new image analysis metrics will increase the clinical utility of AO retinal imaging.

## Figures and Tables

**Table 1.** Cone density estimates in histology and AO retinal imaging studies taken at increasing eccentricities from the foveal center.

Work	Subjects (N.); age (range, years); AxL (range, mm); sampling window area (μm or pixels); model eye	Cone density (average, cones/mm^2^) as a function of retinal eccentricity (range, μm) along the horizontal meridian			
		230–360 μm	400–540 μm	720–890 μm	1,000–1,350 μm
Curcio *et al.*[86]	7; 27–44 yrs; AxL not reported; variable sampling windows; anatomical schematic eye	Nasal/Temp: 60,000–55,000	Nasal/Temp: 40,000	Nasal/Temp: 26,000	Nasal/Temp: 20,000
Li *et al.*[83]	18; 23–43 yrs; 22.9–28.3 mm; adaptive windows (adjusted to contain 150 cones); Gullstrand schematic eye model	All meridians: 60,000–45,000	Not reported	Not reported	Not reported
Chui *et al.*[82]	11; 21–31 yrs; 22.8–27.5 mm; 150 × 150 pixels window; standard reduced eye model	Not reported	Nasal/Temp: 41,000	Nasal/Temp: 27,000	Nasal/Temp: 15,000
Chui *et al.*[84]	4; 24–54 yrs; AxL not reported; 22 × 22 μm window; model eye not reported	Not reported	Temp: 30,000	Not reported	Temp: 15,000
Song *et al.* [85]	10; 22–35 yrs; 22.1–26.1 mm; 50 × 50 μm window; Indiana model eye	Nasal: 59,700–50,000 Temp: 59,200–50,500	Nasal: 43,700–37,800 Temp: 41,200–37,300	Nasal: 29,100–24,200 Temp: 28,100–24,100	Nasal: 19,100–16,800 Temp: 19,900–16,300
Lombardo *et al.* [91]	12 (24 eyes); 24–38 yrs; 22.6–26.6 mm; 50 × 50 μm window; Gullstrand schematic eye model	Nasal/Temp: 49,400	Nasal/Temp: 38,500	Nasal/Temp: 30,000	Not reported

## Supplementary Files

Supplementary File 1 Supplementary Material (AVI, 8288 KB)
